# Implementation Considerations for an Occupational Therapy-Led Mental Health Care Navigation Program for Older Adult Patients

**DOI:** 10.1177/11786329261486980

**Published:** 2026-09-03

**Authors:** Marina Motsenok, Shaghayegh Mirbaha, Mary Clarke, Kristin Morrow, Amanda Knoepfli, Naomi Ziegler, Tracey DasGupta, Sander L. Hitzig

**Affiliations:** 1 282299 St. John's Rehab Research Program, Sunnybrook Research Institute, Sunnybrook Health Sciences Centre, North York, ON, Canada; 2 71545 Sunnybrook Health Sciences Centre, North York, ON, Canada; 3 SPRINT Senior Care, Toronto, ON, Canada; 4 Rehabilitation Sciences Institute, Temerty Faculty of Medicine, 7938 University of Toronto, Toronto, ON, Canada; 5 Department of Occupational Science and Occupational Therapy, Temerty Faculty of Medicine, 7938 University of Toronto, Toronto, ON, Canada; 6 Dalla Lana School of Public Health, 7938 University of Toronto, Toronto, ON, Canada

**Keywords:** mental health, older adults, patient navigation, occupational therapy, continuity of care, implementation

## Abstract

**Background:**

Older adults with mental health (MH) challenges often face significant barriers when navigating complex healthcare systems, especially during critical transitions, such as hospital discharge. These challenges are compounded by comorbid physical illnesses, cognitive impairments, and social determinants of health.

**Objectives:**

This qualitative study aimed to gain insights from patients and providers on factors influencing the implementation of an innovative occupational therapy (OT) led Patient Navigation Program (PNP) supporting older adults with complex MH care needs.

**Methods:**

We conducted a qualitative study that was informed by the Consolidated Framework for Implementation Research. Data were collected through semi-structured interviews and focus groups.

**Results:**

Seven patients and caregivers, and 17 healthcare professionals who served as the MH PNP or collaborated with the MH PNP participated in the study. MH Navigators offered valuable, specialized skills to address gaps in MH and functional outcomes among older adults and were highly effective at bridging hospital and community care. However, the success of such a role depends on alignment across organizations, sufficient professional support, clear role expectations, and integrated team structures. Significant barriers for implementation included organizational cultural differences, system constraints, and the high complexity of patient needs.

**Conclusion:**

The OT-led MH PNP holds significant promise for optimizing care delivery for older adults with MH care needs by bridging gaps in the healthcare system and fostering better continuity of care. Insights from this study can refine navigation processes, enhance interprofessional collaboration, improve patient experiences, and provide valuable guidance for implementing MH navigation programs at other sites.

## Introduction

Older adults with mental health (MH) challenges often struggle to access and navigate healthcare systems, particularly during critical transitions like hospital discharge.^1^ This stage is especially complex for older adults who frequently experience multiple chronic health conditions alongside MH concerns. These comorbidities—such as diabetes, cardiovascular disease, or arthritis—can complicate recovery and contribute to poorer health outcomes.^2,3^ Many older adults also face cognitive and physical impairments, making it harder to manage their care.^4^ Hospital discharge can leave them vulnerable to fragmented care, delayed follow-ups, and readmission, especially when there are challenges in connecting to community resources.^5,6^

MH challenges are common in adults over the age of 50, which may include prevalent conditions such as depression,^7^ anxiety,^8^ dementia,^9^ and substance use disorders.^10^ The co-occurrence of depression and anxiety with chronic physical illnesses is common,^11^ while dementia presents additional challenges by requiring significant caregiving and support.^12^ Substance use disorders are also on the rise, especially for alcohol and prescription drug misuse.^13^ For many older adults, these MH challenges are compounded by social determinants of health, such as low income, insecure housing, challenges with transportation, and social isolation, all of which can make accessing care more difficult.^14^

MH illnesses are multifaceted and complex, requiring tailored approaches rather than a one-size-fits-all treatment.^15^ Their management often involves collaboration among various specialists and healthcare providers.^16^ However, MH care systems frequently lack coordination between providers and facilities.^16,17^ These systems may be geographically dispersed, funded by different entities, and characterized by providers who have expertise in specific areas but limited interaction with other care providers.^18,19^ Additionally, effective treatment for MH conditions often requires a sequential approach, prioritizing the stabilization of conditions that interfere with treatment effectiveness (e.g., urgent comorbidities) before introducing MH interventions,^16^ as well as clear and efficient communication between professionals managing physical and MH.^17^ Compounding these challenges, MH symptoms can sometimes overshadow chronic illnesses, hindering comprehensive care from MH providers.^20-22^

Patient navigation offers a promising approach to addressing these challenges.^23^ Patient navigators guide individuals through complex healthcare systems, helping to connect them with resources, coordinate their care, and remove barriers that might prevent them from receiving timely services.^24^ Although patient navigation has primarily been used in cancer care and chronic disease management,^25^ its value for older adults with MH needs—particularly those transitioning out of hospital settings—is becoming increasingly recognized.^23^

Allied healthcare professionals, such as social workers and occupational therapists (OTs), play a key role in providing transitional care, facilitating smoother adjustments, addressing unmet needs upon discharge^26^ and enhancing functional outcomes.^27^ This multidisciplinary approach is particularly valuable for individuals with co-occurring MH challenges, as it helps mitigate the risk of adverse events during care transitions.^28^ OTs may be especially well-suited for the MH navigation role since they are trained to address the physical, cognitive, emotional, and environmental factors influencing health; therefore, bringing a holistic, client-centred perspective to their work.^29^ OTs help individuals engage in meaningful daily activities, overcome functional limitations, and navigate complex systems of care. For older adults with MH challenges, OTs can bridge the gap between hospital discharge and community reintegration by fostering strong therapeutic relationships, identifying barriers to care, and advocating for appropriate resources.^30,31^

Given the complexity of managing older adults’ MH care needs, a major acute care hospital and community agency in Toronto, Ontario, partnered to expand an existing social work–led pilot patient navigation program (PNP)^32-34^ to support this population across their healthcare journey, including transitions back to the community. This pilot PNP and the subsequent MH expansion are time-limited programs being funded through philanthropy. Although prior work identified key implementation considerations for refining the initial PNP pilot,^35,36^ this expansion introduced substantial changes to the role’s scope and structure, shifting from using community-based social workers to using hospital-based OTs to deliver the program. These changes warranted further evaluation of the implementation. Therefore, the objective of this prospective qualitative study was to examine the organizational and system-level factors influencing the implementation of an OT-led MH PNP for older adults and to identify opportunities to optimize the role.

## Methods

### Study Design

A qualitative^37^ descriptive study design^37,38^ was used to gather insights from patients, caregivers, and healthcare providers (HCPs) who either served as MH patient navigators or interacted with them. This design is appropriate for health services research when the goal is to generate a thorough, practice-oriented account of participants’ experiences. Qualitative methods are especially valuable for exploring complex issues encountered by HCPs and decision-makers.^39^ Data recruitment and collection were conducted between December 2023 and December 2024.

### Study Setting

The study was conducted at a tertiary care academic hospital serving a high-complexity adult population in a major urban setting (Toronto, Canada). The hospital treats over 1.3 million inpatients per year, with a high proportion (nearly 60%) of patients being older adults. To address the growing MH and addiction needs of older adults in the community, the hospital partnered with a local community agency operating within the same urban catchment area, and they developed a novel MH PNP to support older adults across the continuum of care. The local community agency specializes in providing community-based services for older adults, including transportation, home care, and supportive housing, and serves over 4,000 clients per year. The target population were adults aged 55+ who live in the community agency catchment area, and their MH and/or addiction was impacting their ability to live safely and independently at home. This pilot program was based on an existing model of care in which community agency social workers provided healthcare navigation support to older adults with complex needs who were treated at the partnering hospital.^32,33^ As noted above, the funding for these programs is time-limited, as they are supported through philanthropic efforts, with ongoing efforts to secure permanent funding to ensure their sustainability.

In the Fall of 2023, an OT with MH expertise was hired for a full-time position within the hospital to support older adults with MH needs after hospital discharge and provide guidance to patients and their families across various care settings (i.e., from hospital to community). A second OT, with physical medicine expertise, was hired on a part-time basis to support patients’ mobility and home safety needs. Patients were usually referred to and introduced to the navigators by hospital HCPs before discharge, with an intended support period of up to 90 days post-discharge. In practice, however, the complexity of the patient population meant that the duration of support varied widely, ranging from just a few days to several months.^40^ The navigators supported patients by increasing accessibility to community services (e.g., meal services, transportation, financial assistance, and medical care at home), providing support and companionship during transitions (e.g., supportive counselling), and offering home environment support (e.g., providing mobility aids, bathroom equipment and home modifications).

### Participants and Recruitment

Participants included HCPs from the hospital and the community agency, as well as patients who received MH navigation support and their caregivers. Patients and caregivers were eligible to participate in the study if they were adults (≥18 years) admitted to the hospital, or family members of such patients, were fluent in English to complete informed consent, and were able to tolerate one-hour interviews. HCPs were eligible if they worked at the hospital or in a relevant community setting (e.g., community care agency) and had experience of interacting with, or knowledge of, patient navigators. The MH patient navigators were also eligible to participate in the study. All HCPs were required to be fluent in English to complete informed consent and participate in qualitative interviews and/or focus groups.

Participants were identified by the navigator or another member of the hospital team and asked, either in person or via email, whether they would be willing to be contacted by the study team to learn more about the research. After obtaining consent, the first author (MM) contacted potential participants to invite them to an interview. To capture a range of perspectives, purposeful sampling was employed to recruit participants from diverse professions and roles across hospital and community settings.^41^

Six to 12 transcripts are often enough to identify all relevant codes,^42^ and others have found that a 10+3 criterion is a fairly effective guide for setting a priori qualitative sample sizes.^43^ As such, our intended sample size for this study was to obtain interview data from up to 24 professionals and up to 12 patients and/or family members, based on our past experience undertaking similar studies to identify all relevant themes.^44-47^

Our team determined that a sample of 24 interview participants provided sufficient information power,^48^ as evidenced by the alignment with the study objectives, which were to examine the delivery and impact of a MH PNP for older adults, while identifying organizational and systemic factors influencing its implementation. This conclusion was supported by the depth and richness of the interview data, which enabled the identification of common themes.

### Data Collection

Participants took part in at least one interview or a focus group, either in person, on Zoom, or by phone, at home or at their place of work, depending on their preference and availability. Only the interviewers and the participants were present during the interviews. Four participants (three decision-makers and one MH Navigator) were interviewed a second time, approximately 11-12 months after their initial interview, to reflect on how their experiences with the MH PNP changed over time. Three focus groups, 17 individual interviews and one patient and caregiver dyad interview were conducted in total. The interviews were conducted by the first author (MM), a female Postdoctoral Fellow, and the senior author (SLH), a male Senior Scientist, both of whom hold PhDs and are trained in qualitative research methods. Interviews followed a semi-structured interview guide (see Appendices A and B). MM had no prior relationship with the participants. SLH had previously engaged in professional interactions with three professional leaders who participated in a focus group. All participants were informed that the interviewers were conducting the interviews in their roles as hospital-affiliated researchers with research interests in older adult care and healthcare services. Interviews were audio-recorded and transcribed verbatim. Interviewers took notes during the interviews to facilitate them. Patients and caregivers were informed ahead of their interviews that they would receive a $25 gift card as a token of appreciation for their time.

Participants were interviewed on several key topics, including: a. their understanding of the role of MH patient navigators; b. their experiences working/receiving support from the navigators; c. what they found to be most helpful about the navigation program; and d. suggestions for improvements. HCPs were also asked to discuss their perceptions of the role and the reasons for its creation, as well as organizational and system-level factors affecting the implementation of the MH patient navigation role. Additionally, they were asked to consider how a similar program could be implemented under ideal conditions and the unique qualities OTs bring to this role. Interviews were tailored to each participant’s area of expertise and conducted with consideration for their time constraints. The interview guide was informed by the Consolidated Framework for Implementation Research (CFIR),^49^ which has been widely used to study key implementation considerations across various health interventions,^34,35^ and is well-suited to prompting participants to reflect on factors that facilitate or hinder the implementation of the MH patient navigator role.

### Data Analysis

Data was analyzed using an inductive thematic analysis approach as outlined by Braun and Clarke.^37^ This process involved open coding and creating categories based on patterns in participants’ interview transcripts.^37^ The data analysis commenced during the data collection period. The first and second authors (MM, SM) reviewed the transcripts while working with the senior author (SLH) to establish the coding framework. All data were coded using NVivo 14.^50^ The themes were finalized, named, and defined through collaboration among authors (MM, SM, SLH). A detailed audit trail was created to document the criteria used to assign data to each theme. Analytic rigour was strengthened through triangulation across multiple analysts, regular team meetings, and ongoing reflexive discussions about potential biases and experiences that could shape data interpretation. We used a shared codebook to ensure coding consistency and included representative participant quotations to enhance transparency and deepen understanding of the findings. The study also adhered to the Consolidated Criteria for Reporting Qualitative Research (COREQ) reporting guidelines.^51^ Once the data were analyzed, preliminary findings were shared with decision-makers and providers involved in the PNP to obtain their feedback. Participant feedback did not identify any concerns or require substantive changes to the interpretation of the findings. To describe key findings, illustrative quotes from the interview transcripts are provided in the results section. However, to preserve the anonymity of our participants, only details about their practice location (hospital versus community) are provided.

## Results

We conducted interviews with 17 HCPs (see Table 1 for HCPs’ characteristics), as well as five patients and two family caregivers. The mean age of patients and caregivers was 69.6 (range 59-77), and 57.1% were women. Three themes were identified: a) Navigating practice and system-level tensions and expectations; b) MH OT characteristics and services, and; c) collaboration for a path forward (see Table 2 for themes and sub-themes). A pervasive theme that influenced the design and rollout of the MH patient navigator role was the *shared history* between the hospital and community agency as well as the unique professional experience of the MH OT navigator who had pre-existing employment within the hospital, and who interacted with community programs and agencies over the years.

*“Having an internal team member that is already really aware of the mental health system within the hospital, that's been huge with navigating certain programmes, making connections to other team members in the hospital.”* (MHS1, decision-maker, community agency).

**Table 1.** Overview of HCPs Characteristics (n = 17)

Characteristic	n (%)
Gender	
Women	16 (94.1)
Men	1 (5.9)
Organization	
Hospital	10 (58.8)
Community agency	7 (41.2)
Professional Role	
Social worker	7 (41.2)
OT	3 (17.6)
MH Navigator (trained as OT)	2 (11.8)
Decision Makers	5 (29.4)
Years of Experience in the Current Role	
Less than one year	4 (23.5)
1-4 years	4 (23.5)
5-9 years	5 (29.4)
10 or more years	4 (23.5)

**Table 2.** Themes and Sub-themes

Pervasive theme	Themes	Sub-themes	Illustrative quotes
Shared history	Navigating practice and system-level tensions and expectations	Expectations and real-world roll-out	*“I think it goes back to the liability side. And specifically, when we were doing documentation, there was concerns from the employee around the liability of documenting in a separate system. We have our own system and the hospital has a separate system. That became a concern pretty early on. And I think the documentation was inconsistent because we were using two different systems.”* (MHS1, Decision-maker, community agency).
		Demand, patient complexity and limited resources	*“This isn’t just [city] Province kind of thing. This is a world problem, that there is a significant gap in mental health long-standing support. This type of client … need lifetime support. It doesn’t have to be as intensive, but they need to be able to have that touch base. (…). At the end of the day, this is a huge gap in care for this population.”* (MHS6, Decision-maker, community agency).
		Adaptation to tensions	*“And so that’s why we do have weekly meetings with [MH navigator] from the [hospital] side to hear how things are going, to remove some of those tensions, through our talking together behind the scenes with the leaders, so that we can free our clinical staff to do the clinical work.”* (MHS9, Decision-maker, hospital).
	MH OT characteristics and services	​	*“Because we’re looking at the environment and the person in it, and the activities that they want to be able to achieve (…). They might have better coping strategies in a familiar setting and things like that. And then I do think that we look at the whole person. And for myself, I’m able to help them with funding for equipment. Even if there’s somebody else that needed modifications to his assisted living, and the social worker wouldn’t have been able to fill it out, it had to be by an OT. So I think in certain cases it’s helpful to have occupational therapy involved.”* (MHS10, MH Navigator, hospital).
	Collaboration for a path forward	​	*“(…) in terms of integrated and collaborative care. So it’s not just collaborating, it’s actually integrating. So having another professional lens as part of the team in an integrated way, it means we learn from each other and share each other’s skillsets. Which is, from where I’ve worked previously, that’s always been such a beneficial part of the work as an inter-professional team.”* (MHS8, MH Navigator, hospital).

While this shared history facilitates the creation of the role, it has also led to expectations that were not fully realized due to practice and system-level considerations absent in the existing PNP, which uses community agency social workers embedded in hospital settings. Due to regulatory constraints, the community agency was only permitted to hire social workers and could not employ other allied health professionals. As a result, the OT navigators were hired and managed through the hospital. Community agency leaders identified integrating these hospital-based OTs into their organization as one of the most significant implementation challenges. Differences in organizational culture, such as role flexibility and professional hierarchy, created tension and friction, as there were legal challenges to exchanging information between the two organizations. This led to a different roll-out compared to the pre-existing social-work-led PNP. When considering future adjustments to the role, one community agency leader suggested that having OTs employed directly within the community setting might support stronger collaboration:

*“I think in the learnings and what might work out differently is we were aware or are aware of the differences in a community lens on healthcare and a hospital lens on healthcare and how different they are. And it is a bit oil and water. And I think the same discipline from a community organization (…) that coming into this project would have a more aligned approach to how we provide client care.”* (MHS5, Decision-maker, community agency)

However, the shared history facilitated ways to navigate some of these challenges, fostered provider positivity and trust towards the role, and led to a number of benefits for patients with complex MH needs, such as timely access to MH resources while providing “*a very helpful safety net for the patients.”* (MHS17, HCP, hospital). Several decision-makers commented on the benefit of having an experienced hospital employee serve as the navigator, as they had existing relationships with hospital staff and could navigate the hospital environment effectively to benefit their patients.

“*Well, I think for the mental health OT especially, she’s got experience working on [name] team. So actually, with individuals who frequently access the hospitals. She comes in with that expertise of both community as well as hospital. So we were looking for that. Somebody that knew how to navigate the hospital system in terms of the different mental health resources that we're not as aware of. The OT knows a lot about it. She knows the team members there. She knows the in-person psych unit. She knows the emergency department. So that was a huge value-add for us too. Because we don’t have to navigate those services in the hospital.”* (MHS1, Decision-maker, community agency).

Overall, the MH navigator’s familiarity with both hospital and community settings was extremely valuable in navigating the challenges posed by the new role configuration.

### Navigating Practice and System-Level Tensions and Expectations

This theme and accompanying sub-themes describe how practice and system-level considerations influenced the roll-out of the novel MH navigator role. Broadly, the main theme centers on how initial expectations for how the role could enhance the existing PNP shifted in the face of factors related to the scope of practice of a hospital-based OT, interactions with the partnering community agency, patient demand and complexity, and system-level considerations (i.e., catchment area). The sub-themes of *‘expectations and real-world roll-out’, ‘demand, patient complexity and limited resources*’, and *‘adaptation to tensions’* all describe how they shaped the current MH navigator role.

#### Expectations and Real-World Roll-Out

The existing PNP for older adults used community agency social workers who were embedded in the hospital. However, the decision to select a hospital-based OT to work as an MH navigator with the partner community agency was informed by scope of practice considerations. The designers of this model initially thought these considerations would accelerate the discharge process. Several participants repeatedly emphasized the perceived value of the OT’s expertise, as one community social worker explained that the hospital-based OT *“could bring a whole different aspect of how she’s worked with certain psychiatrists. (…) She understands the medication and the effects. It’s just a whole different level of expertise. So that’s really important.”* (MHS4, HCP, community agency).

While some of the benefits of hiring an OT, in terms of their expertise and clinical training, were realized, the scope of practice and institutional considerations led to some initial expectations not being fully met. Hiring OTs for the MH navigator role introduced additional layers of complexity, as social workers and OTs practice under different legislation (the Regulated Health Professionals [RHP] Act^52^ vs. the Social Workers and Social Service Work [SWSSW] Act).^53^ These differences influence documentation requirements and approaches to patient care, further compounding the existing distinctions between hospital-based and community-based practice, as one decision-maker stated: *“liability is a lot heavier in a hospital. I think that it's just overarching of their care (…) They are just a lot bigger. It’s a big organization. And it comes with a lot more red tape.”* (MHS5, Decision-maker, community agency). Several participants noted that the role’s clarity has evolved over time, and that the MH navigators themselves often had to articulate the scope of their work and recalibrate expectations in light of professional regulations. Additionally, the navigators were generally more experienced, mid-career clinicians, whereas community agency social workers were typically earlier in their careers. Therefore, the navigators experienced limited opportunities for peer support while expecting greater professional autonomy.

Although there was a perception that prior collaborations between the organizations would lead to a smooth implementation, participants expressed that disparities in documentation systems, professional regulations, policies and role expectations became apparent and impacted their relationships. However, this issue is not unique to these sites, as the RHP Act has specific documentation requirements that limit where clinical notes can be stored, making it a system-level challenge that affects many inter-agency collaborations. While the community agency staff and navigators could access each other’s documentation systems to review clinical notes when needed to facilitate care plans, the constraints imposed by the RHP and SWSSW acts contributed to a sense of disconnect between the two organizations, particularly in communication, culture, and infrastructure.

#### Demand, Patient Complexity, and Limited Resources

The volume of MH support needs for older adults, along with their complexity, led the program to provide higher-intensity, longer-duration services than initially anticipated, which strained MH navigators’ bandwidth. One participant noted that:

*“Definitely this is something that I think we missed, is that the level of support and duration is so important for mental health population. And that was, I remember, a gap even from the beginning that was communicated, was, how is there an expectation to only follow these clients for 90 days when there's just so much there?”* (MHS1, Decision-maker, community agency).

In response to patients’ needs in physical medicine and rehabilitation, and delays in receiving these services from provincial providers, a second OT was hired for the MH navigation role to focus on supporting patients’ physical functioning and independence in the home environment, thus complementing the MH component of the role.

*“There were two OTs hired for this role. One was a mental health OT specialist and one was a physmed [physical medicine] OT. They are two very different focuses. And the physmed OT was really due to a gap of service from our larger service provider, [organization], because the delays were so long. And that was prohibiting functionality back in their home environment. That’s where that focus lied. The mental health OT (…) brought a different type of speciality service that [community agency] and other service providers were not able to provide.”* (MHS6, Decision-maker, community agency).

Several participants also highlighted the limited availability of long-term MH support as a significant gap in care for the target population, expressing concern that limited resources place pressure on both navigators and the broader healthcare system, creating an unsustainable navigator role under the current model. There was a general sense that the health system was under-resourced to meet complex MH needs, which contributed to greater reflection on what a clinically meaningful, yet pragmatic, length of service should look like.

#### Adaptation to Tensions

All of these issues created points of tension that required the key interest groups to navigate patient, practice, and system-level challenges, which in turn adjusted their expectations for how the MH navigator role could operate. These points of tension were initially due to the need to gain a better understanding of RHP Act, which was believed to be a misalignment between the cultures of the hospital and community, with one decision-maker from the community stating:

*“Especially like what I was mentioning earlier about the liability and the risk, assessments are different for facilities and for the community. And it's not that one is right or one is wrong. They’re just different. And that's a lot of misalignment. And I guess that there wasn't an opportunity to really converse about, how do we overcome some of these unforeseen risks or liabilities.”* (MHS1, Decision-maker, community agency).

Thus, there were some limits that both teams did not initially expect when designing the MH PNP, but that became apparent once the program was rolled out.

Despite the early tension and changed expectations, the novelty of the MH navigator role afforded a degree of flexibility to meet the MH needs of patients, and an opportunity for the MH Navigator to build on their clinical experience to help shape the role: *“…it’s actually just been up to me to define the role and my expectations”* (MHS8, MH Navigator, hospital). In some cases, the pre-existing ties the MH navigator had with staff and knowledge of hospital and community agency cultures were useful in navigating towards solutions, even if not in a way that aligned with early expectations. The relationships between hospital and community agency leaders facilitated mitigating some of these tensions through mutual communication and weekly meetings.

### MH Navigator Characteristics and Services

This theme highlights the experience and unique qualities that the MH navigators brought to the role, enabling them to effectively address the MH needs of older adults participating in the program. It also outlines the specific services provided and the ways in which care was delivered. Participants often described the role as closing critical gaps between the hospital’s services and the community agency’s services, such as bringing MH resources to the community and enabling longer, more in-depth MH interventions. For instance, one participant noted:

*“(*…*) accessing mental health resources is not easy. There’s a long wait list for mental health case managers. So having an in-house mental health person is extremely helpful for our team to even jointly work together to support the client that we’re noticing.”* (MHS1, Decision-maker, community agency). While another stated:

*“So I think that she was able to, when it was more complex around the mental health piece and functioning, that she had more ability to do more in-depth interventions, which for me gave me peace of mind. Because I knew that, for example, some of the patients I saw, I couldn't address their needs within two to three visits.”* (MHS17, HCP, hospital).

Participants described how the MH navigator role extended well beyond counselling and service coordination to include home safety assessments, functional evaluations, and assistance with equipment funding applications. These responsibilities fell outside the typical scope of social work practice, underscoring the distinctive skills and contributions that OTs brought to the role.

*“(…) from a community standpoint, that is one of the very big barriers. You require certain training, and this is an occupational therapist that would normally do this to assess for equipment and then apply for specific funding. From a social work perspective, we can look and identify that somebody’s mobility is not good, but not give the equipment.”* (MHS1, Decision-maker, community agency).

Participants further emphasized that the MH navigators’ person–environment–occupation focus made them particularly effective in supporting older adults with MH needs, noting that *“it is really helpful to be able to go and see somebody in their home environment and see how they are managing there”* (MHS10, HCP, hospital). This approach helped older adults develop better coping strategies within their familiar living environments and promoted a more holistic model of care.

### Collaboration for a Path Forward

This theme emphasizes that collaboration is a critical factor in the program’s success and that optimizing the MH navigator role requires a team-based approach. Strengthening collaboration would create opportunities for continued professional development and clarification of how MH navigation functions—both as a distinct role and as part of a broader multidisciplinary team. Ultimately, this would support better integration of care, particularly during the transition period after patients are discharged from the MH navigation program.

An integrated model of care enables earlier identification and more holistic management of MH concerns. Professionals described how bringing together diverse perspectives—particularly the combined expertise of OTs and social workers—can facilitate proactive responses to emerging issues and enable more tailored, coordinated supports. As one decision-maker elaborated:

*“I definitely think if we're taking in increase in complexity, we also need to complicate the lens in which we're viewing these clients. So offering different perspectives like a mental health OT is critical. We can't just be viewing these clients in the traditional sense. We need to expand our clinical skills. So [hospital] has offered this amazing opportunity to partner with allied health members, occupational therapy is just a logical next step. We're looking at their practical environments. Like it complements the skills of social work so well, they partner very, very frequently in the community.”* (MHS5, Decision-maker, community agency).

Several HCPs highlighted the instrumental partnership between social work and OT and the importance of collaboration within interprofessional teams moving forward. For example, a hospital-based social worker noted:

*“(…) it’s instrumental to have both a social worker and an occupational therapist, if there was ever room to expanding this programme, because I do think that we all have our expertise in different areas. I think it’s important that we’re working from a collaborative perspective, I don’t think it should be isolated to one profession. I think there is room and opportunities for many professionals to be participants in a care plan for an individual. Often an occupational therapist would be limited with a person who might be immobilised due to their mental health and their ability to move, for example, and I’m sure we would refer to a physiotherapist. I think the mental health component is the important aspect of this role in terms of their expertise and their knowledge. And whether or not you hire an occupational therapist, a social worker or a physiotherapist, I think there is room to expand that programme to encompass a team.”* (MHS16, HCP, hospital).

One of the challenges identified by the MH navigators and decision-makers was the sense of isolation experienced in the role, stemming from limited opportunities for professional guidance and support—a challenge that was further amplified when the second navigator’s contract was not extended beyond the first year, prior to the completion of the pilot:

*“I don't know if it's experience or just that she's also an OT, but often I use her as a sounding board, even with clients she's not working with me on. But then, with the clients we're working on together, it's just been so great. Having that sounding board and that sometimes validation, sometimes reality check on my expectations, it translates to other patients that I'm working with too. So, having her leaving is a huge loss for me. I'm feeling like it's going to be quite isolating, going forward for me, in the role.”* (MHS8, HCP, hospital).

Therefore, one of the considerations moving forward was a collaborative model in which the MH navigators are part of an inter-professional team:

*“There's been a lot of learnings about the needs and the challenges. (…) We might not have the guidance that this person is needing and the support on our team because (…) their direct support comes from an external agency who maybe doesn't have the capacity to even give that. It leaves them feeling a bit siloed. If we're putting them on a caseload and they're needing professional practice support and guidance, is that really the best practice for the clients and for the individual to feel that they're supported properly? Whereas in a consulting role, I think this alleviates a lot of that pressure. And we could maybe work a little bit more collaboratively. I definitely think that's a consideration. (…)I think they were missing that gap in not feeling like they had someone to be talking to about the cases, to be getting their own direction. Because as much as they're an expert, they also deserve to have leadership. They're doing front line work. And we need to be providing that to them.”* (MHS5, Decision-maker, community agency).

Overall, several critical reflections were shared about the structure of the MH navigator and the types of organizational supports needed to ensure they can operate in a manner that provides clear direction and fosters greater cohesion with the teams managing and working in the various PNPs.

## Discussion

This study examined the implementation of a novel OT-led MH PNP for older adults with MH needs who transition from hospital to community settings. Three themes shaped participants’ experiences with the program: navigating practice- and system-level tensions and expectations; the characteristics and services of MH Navigators; and collaboration toward a path forward. These themes highlight both the promise and challenges of integrating specialized MH navigation into existing models of care for older adult patients.

A central finding was the influence of structural and organizational factors on role implementation. Although the program leveraged a long-standing partnership between the hospital and the community agency, regulatory constraints prevented the community agency from hiring OTs directly. As a result, MH OT Navigators were employed and supervised by the hospital while working in a community setting. This structure introduced unanticipated complexity related to documentation and the scope of practice. Differences in organizational culture and expectations for professional autonomy further contributed to friction. When framed within the CFIR, these findings highlight the importance of cosmopolitanism, which describes how well two separate organizations are integrated to deliver an intervention. While both the hospital and community agency had established a degree of trust over the years,^35^ there were still some ongoing tensions that required further navigation to optimize the MH OT role. Prior work emphasized that navigation roles often operate across institutional, sectoral, and geographic boundaries and therefore require clear governance structures, shared documentation processes, and explicit role expectations.^34^

Despite these challenges, the MH navigators’ clinical expertise and vast experience emerged as a significant facilitator for patients’ successful healthcare transitions. Their training enabled them to provide functional assessments and subsequent patient-centred interventions that were not available through an existing patient navigation model.^32^ Participants described the benefit of navigators’ ability to conduct home safety evaluations and equipment funding applications, which addressed critical gaps in community care. This outcome aligned with the original vision for the role,^54^ providing additional evidence for the validity of the role and aligning with the CFIR’s Intervention sub-construct that highlights how the underlying evidence of an intervention may influence uptake. These findings are consistent with the literature describing OT’s contribution to transitions and healthy aging through assessment of function, participation, routines, environmental barriers, and community participation.^55^ They also align with emerging evidence that OT competencies map closely onto navigation functions, including communication, equity-oriented practice, and engagement across systems of care.^54^

The navigators’ familiarity with hospital systems and pre-existing relationships with hospital staff were key enablers of timely access to psychiatric care. These advantages underscore the unique role of navigators in bridging functional and MH needs in supporting transitions in care for older adults with MH challenges. Again, when framed within the CFIR, the use of the OT highlights the innovation’s relative advantage (CFIR Intervention) for the MH older adult patient population. Additionally, it highlights how individuals in MH roles were confident in their ability to perform their roles (CFIR Individual Domain, innovation deliverers), even when MH navigators needed to provide leadership in defining certain aspects of their scope of practice.

However, high patient complexity and limited MH resources in the community led to service demands exceeding program capacity. The 90-day follow-up expectation proved misaligned with the chronic and multifaceted nature of many patients’ MH needs. The limited availability of long-term MH supports for older adults left navigators in a challenging position, managing enduring patient needs without sufficient community programs to facilitate a smooth transition and ensure continuity of care. As such, the patient needs and resource limitations (CFIR Outer Setting) were far more complex than initially planned, and this requires further reflection on how to optimize the delivery of the role.

The study also illuminated the importance of inter-professional collaboration in supporting effective MH navigation. Participants described strong potential for integrated models in which OTs, social workers, and other allied health professionals work collectively to identify concerns early, tailor interventions, and share expertise. However, the siloed nature of the navigator’s role, exacerbated by the departure of the second navigator, resulted in professional isolation and limited opportunities for clinical support. This finding suggests that even when a navigation role is clinically appropriate, its implementation may be weakened if it is not embedded within an interprofessional infrastructure that supports supervision, shared decision-making, and workload distribution.^56^

Taken together, our findings contribute to the growing body of evidence on PNPs for older adults^32-34,36,54,57^ and extend this literature by examining an OT-led model focused specifically on MH transitions. Existing studies of patient navigation for older adults have highlighted the importance of role clarity, relational continuity, timely information, caregiver support, and assistance with accessing health and social care resources.^55,58^ Our findings build on this work by suggesting that OT-led MH navigation may be especially valuable when older adults’ MH needs intersect with functional decline, environmental barriers, safety concerns, and difficulty re-establishing daily routines after discharge. Critically, the current MH PNP, along with its predecessor,^32,33^ is viewed as a pilot program, as it is funded by philanthropic mechanisms with a targeted end-date in 2026-27. As such, the evidence collected from this study can inform hospital and community decision-making on the impact of these programs and ways to improve their delivery, thereby contributing to strategies for their ongoing sustainability.

Several implementation considerations emerge from this study. First, PNPs would benefit from aligning employment structures, and organizations would benefit from developing a clear understanding of the documentation policies that result from membership in different regulated health professions and settings, to reduce administrative burden and clarify expectations. Second, embedding MH navigators within an interprofessional team may mitigate isolation, enhance clinical decision-making, and support. Additional opportunities for clinical supervision would also be beneficial to address some of these concerns. Third, programs should consider more flexible service duration and clearer pathways for stepped or ongoing MH support. Finally, the strong complementarity between OT and social work suggests that team-based navigation models, rather than single-provider models, may be more effective for supporting older adults with MH needs.

## Limitations

Qualitative research aims to provide a deep understanding of specific phenomena by exploring individual cases in depth rather than making broad generalizations.^59^ Therefore, our findings offer insights specifically into the experiences of patients, caregivers and providers at a large metropolitan centre in Ontario, Canada. Although we interviewed patients, caregivers, and providers and analyzed all interviews together, this manuscript focuses primarily on provider perspectives, as they reflected factors influencing the implementation and delivery of the MH navigator role. While patient and caregiver perspectives provided valuable insights into the program’s impact, they are underrepresented in the manuscript, as their accounts primarily centred on care experiences rather than program implementation.

## Conclusion

The complexity of addressing MH issues in older adults underscores the importance of innovative approaches like the MH PNP. This study highlights the promise of an OT-led MH PNP by integrating system coordination with functional, environmental, and occupation-focused assessment and intervention. However, successful implementation depends on clear role definition, aligned organizational structures, and inter-professional collaboration. Future studies are warranted to better determine the impact of an OT-led MH PNP on patient outcomes and clinical efficiency, thereby highlighting its contributions to the care of older adults. Regardless, our findings reinforce the need for implementation planning that addresses the organizational infrastructure and interprofessional relationships required to sustain complex transitional care innovations.

## Supplemental Material

Supplemental material - Implementation Considerations for an Occupational Therapy-Led Mental Health Care Navigation Program for Older Adult Patients Supplemental material for Implementation Considerations for an Occupational Therapy-Led Mental Health Care Navigation Program for Older Adult Patients by Marina Motsenok, Shaghayegh Mirbaha, Mary Clarke, Kristin Morrow, Amanda Knoepfli, Naomi Ziegler, Tracey DasGupta, and Sander L. Hitzig in Health Services Insights.

Supplemental material - Implementation Considerations for an Occupational Therapy-Led Mental Health Care Navigation Program for Older Adult Patients Supplemental material for Implementation Considerations for an Occupational Therapy-Led Mental Health Care Navigation Program for Older Adult Patients by Marina Motsenok, Shaghayegh Mirbaha, Mary Clarke, Kristin Morrow, Amanda Knoepfli, Naomi Ziegler, Tracey DasGupta, and Sander L. Hitzig in Health Services Insights.
