# Supplementary material for: Implementation Considerations for an Occupational Therapy-Led Mental Health Care Navigation Program for Older Adult Patients
Source: Health Serv Insights. 2026 Sep 3;19:11786329261486980. doi: 10.1177/11786329261486980 (PMC13546429; doi:10.1177/11786329261486980)
Supplement: Supplementary file 1 [file sj-pdf-1-his-10.1177_11786329261486980.pdf]

PATIENT AND FAMILY INTERVIEW QUESTIONS ON COMMUNITY PATIENT NAVIGATION

1. Please tell me a bit about how things have been going with you (and your family member) and your health since your initial visit at Sunnybrook where you first interacted with the patient navigator.
    a. *How have you (your family member) been feeling? How has this changed from the first hospital visit?*
    b. *Have you (family member) been back to the hospital? If yes, how was this experience difference then the first?*

2. When thinking about your 'journey'to have your (family member's) health and social care needs met since being back in the community, what three words would you use to describe the experience?
    a. *Please explain why you chose those three words.*

3. Please tell me about your experiences with the navigator after you (your family member) went back home?
    a. *What kinds of supports or information did they provide (to you and your family member)? What help or support did you not receive but wish you had received? (Information? Emotional support? Somebody doing something for you? how did you receive that help?)*
    b. *When, how and where did you interact with the navigator? Did this change at all over time?*
    c. *What informaiton about the navigator did you feel was missing?*

4. What was it about the patient navigator that helped you (and your family member) since leaving the hospital?
    a. *What did you (or your family member) find useful about having access to the navigator?*
    b. *How did you feel the navigator worked with other professionals in the community providing care to you (your family member)? Was there any professional or service you were not connected to but wish you were?*
    c. *Was there any thing that did not help you? Why do you think these were not helpful?*
    d. *What aspects of your personality or life experiences do you think influenced your experience with the navigator? How did this influence your need for support? (e.g, Sense of humour? Professional training? Religious/spiritual beliefs? Finding meaning e.g. making sense of illness? Self-care e.g. meditation, regular exercise etc?)*

5. What parts about the patient navigator do you think could be improved upon? What other things could the patient navigator have done differently to make your (family members') transition back to the community?
    a. *Were there things you were hoping the navigator could do for you but didn't?*

b. *What advice would you give to other patient navigators working with family members and patients?*

6. Is there anything else about your time with the patient navigator that you would like to share that I didn't ask you about?
