# Supplementary material for: Implementation Considerations for an Occupational Therapy-Led Mental Health Care Navigation Program for Older Adult Patients
Source: Health Serv Insights. 2026 Sep 3;19:11786329261486980. doi: 10.1177/11786329261486980 (PMC13546429; doi:10.1177/11786329261486980)
Supplement: Supplementary file 2 [file sj-pdf-2-his-10.1177_11786329261486980.pdf]

Staff Interview Guide

1. Please tell me about your role and how it interacts with the mental health navigation program.
    a. *What is the scope of your practice?Does this role involve palliative care in acute care, in the community or both?*
    b. *What kind of supports do your provide related to helping patients/clients and family members navigate the healthcare system?*
    c. *Do you have any recent prior experience at other institutions with health system navigation?*

2. What do you know about the patient navigator role at Sunnybrook?
    a. *What do you know about their scope of practice?*
    b. *What impact, if any, do you forsee the navigator having?*

3. Why do you think that the mental health navigator role created?
    a. *What are the needs that this role aims to address?*
    b. *How does it complement other navigator roles?*
    c. *What are the unique attributes of this role/program?*
    d. *What is currently not being addressed?*

4. How do you perceive the mental health navigator role?
    a. *What kind of support does this role provide for helping patients and family members navigate the healthcare system?*
    b. *What about the mental health patient navigator do you feel is helpful in supporting patients/clients? What do you feel is unhelpful?*

5. Who are the patients that the navigator is supporting/you are referring to the navigator?
    a. *How do you identify them?*

6. Please tell me about your experiences with the navigator (referring staff)?
    a. *When, how and where did you interact with the navigator (referring staff)?*
    b. *What kinds of supports or information did they provide to your patients/clients and/or their family members?*
    c. *What information about the navigator did you feel was missing?*

7. What are the unique qualities that OTs bring to this role?

8. How would you define this program's success?
    a. *How do you think we should measure success? (decrease in hospital visits/stays, reduction in symptoms, improvement in quality of life, engagement with services)*

9. What are the barriers you are experiencing in the implementation of this role?
    a. *What are the facilitators?*

10. What parts about the patient navigator do you think could be improved upon? What other things could the patient navigator have done differently to enhance your patients or family members journey of care?
    a. *Were there things you were hoping the navigator could do but didn't?*
    b. *What aspects of your organization and/or others could be improved to support the navigator role and your own scope of practice?*

11. If money and resources were no object, what would the 'ideal' patient mental health navigator role look like to you?

12. Is there anything else you want to share about the mental health navigator role?
